# The impact of natural disasters on the spread of COVID-19: a geospatial, agent-based epidemiology model

**DOI:** 10.1186/s12976-021-00151-0

**Published:** 2021-12-03

**Authors:** Maximillian Van Wyk de Vries, Lekaashree Rambabu

**Affiliations:** 1 Department of Earth & Environmental Sciences, University of Minnesota, 301-25 Tate Hall, University of Minnesota, 116 Church St SE, Minneapolis, MN 55455 USA; 2 St Anthony Falls Laboratory, University of Minnesota, 301-25 Tate Hall, University of Minnesota, 116 Church St SE, Minneapolis, MN 55455 USA; 3 National Health Service Tayside, Dundee, UK; 4 Edinburgh Medical School, University of Edinburgh, Edinburgh, UK

**Keywords:** COVID-19, Epidemiology, Agent-based model, Natural disaster

## Abstract

**Background:**

Natural disasters and infectious diseases result in widespread disruption to human health and livelihood. At the scale of a global pandemic, the co-occurrence of natural disasters is inevitable. However, the impact of natural disasters on the spread of COVID-19 has not been extensively evaluated through epidemiological modelling.

**Methods:**

We create an agent-based epidemiology model based on COVID-19 clinical, epidemiological, and geographic data. We first model 35 scenarios with varying natural disaster timing and duration for a COVID-19 outbreak in a theoretical region. We then evaluate the potential effect of an eruption of Vesuvius volcano on the spread of COVID-19 in Campania, Italy.

**Results:**

In a majority of cases, the occurrence of a natural disaster increases the number of disease related fatalities. For a natural disaster fifty days after infection onset, the median increase in fatalities is 2, 59, and 180% for a 2, 14, and 31-day long natural disaster respectively, when compared to the no natural disaster scenario. For the Campania case, the median increase in fatalities is 1.1 and 2.4 additional fatalities per 100,000 for eruptions on day 1 and 100 respectively, and 60.0 additional fatalities per 100,000 for an eruption close to the peak in infections (day 50).

**Conclusion:**

Our results show that the occurrence of a natural disaster in most cases leads to an increase in infection related fatalities, with wide variance in possible outcomes depending on the timing of the natural disaster relative to the peak in infections and the duration of the natural disaster.

**Supplementary Information:**

The online version contains supplementary material available at 10.1186/s12976-021-00151-0.

## Introduction

As of July 2021, COVID-19 has spread to over 200 countries, infected more than 188 million, and killed over 4 million people [1]. Natural disasters can threaten the measures in place to reduce disease transmission [2]. Understanding how disease spread can be worsened by natural disasters will aid preparedness and response planning [3]. Although a small increase in risk of epidemic outbreak following natural disasters has been identified [2, 4], very little is known about the spread of infection following a natural disaster during a pandemic such as COVID-19.

Numerical modelling is an important tool for understanding and projecting the spread of infectious diseases [5, 6], including COVID-19 [7–9]. A common approach is to divide a population up into susceptible, infected, recovered and deceased individuals [5, 6, 10]. More sophisticated models incorporate a larger number of different states (e.g. asymptomatic or seriously ill individuals [7];), and may account for other real world complications (e.g. age, vaccination campaigns, etc.).

The time varying proportion of each state may be calculated deterministically by solving a series of ordinary differential equations [5–7]. This approach can accurately portray the spread of infectious diseases on a large scale [6, 7]. The deterministic approach is ill suited for simulation of small population sizes, heterogeneous populations and threshold behaviour [9, 11]. A stochastic, agent-based model structure may also be used [10, 12–14], in which case the variation between runs allows for quantification of uncertainty [8, 14]. Agent-based models are are well suited to represent heterogeneous or complex populations and spatial variability [10, 12, 13].

## Objectives

The main objective of the study is to evaluate the impact of a natural disaster on the spread of COVID-19 infection.

The sub-objectives are the following:

1. To evaluate the impact of timing of natural disaster on infection spread and number of deaths.
2. To evaluate the impact of duration of natural disaster on infection spread and number of deaths.

## Methods

### Spatial, agent based model

We use geoSIR, a geospatial, agent-based model structure to account for the spread of disease and movement of individuals within the model space from a natural disaster evacuation [10, 12]. This model builds on the basic susceptible, infected, recovered model structure to better account for the characteristics of COVID-19 [5, 6, 10]. Every cell in the model is either occupied by an individual, or by empty space (*X*_*e*_). Each model individual may be in one of 7 states: susceptible (*S*), infected and mildly ill (*I*_*m*_), infected and severely ill (*I*_*s*_), infected and asymptomatic (*I*_*a*_), recovered (*R*), or deceased (*D*). Empty space allows us to account for geographic factors such as spatially varying population density and the presence of cities. Individuals may also self-isolate or be quarantined. We base the definition of our states and model disease characteristics on COVID-19 data [15–21], and our model space on real-world geospatial data.

In geoSIR, the spread of the disease is initiated with the arrival of infected travellers [22]. The disease then spreads further through encounters between individuals within the model space. In each timestep, individuals make both close and distant encounters with other individuals. If either induvial is infected, they have a given probability of transmitting the infection to the other individual, which we parametrise based on available data for COVID-19 [20, 23]. We also ensure that resulting reproductive number *R*_0_ (as calculated from eq. 6) are consistent with the range estimated for COVID-19 [15, 19, 24].

Infections may be either asymptomatic, mild or severe. Asymptomatic and mildly infected individuals merely transmit the infection, while severely infected individuals are at risk of death. If the total number of severely infected individuals exceeds the local hospital capacity (for which we use the number of intensive care unit beds), the case fatality rate of severely infected individuals is increased. Further description of the model setup and key equations are provided in the supplementary materials, as well as a brief use manual for the open-source code.

### COVID-19 data sources

We use published data for the spread of COVID-19 to parametrise the disease transmission in our model [15–21]. Our data sources consider the original SARS-CoV-2 strain and not any of the later variants.

The spread of the disease may be described by a disease spread parameter *α*:

1 $$\partial I\left(t+1\right)=\alpha\left(t\right)\overline I\left(t\right)$$

In which I is the number of infected individuals and *α *is given by:

2 $$\alpha (t)=\rho (t)\left({n}_{ci}(t){P}_{ci}(t)\right)+\rho (t)\left({n}_{di}(t){P}_{di}(t)\right)$$

With *n*_*ci*_ being the number of close encounters, *P*_*ci*_ being the probability of infection for close encounters, *n*_*di*_ being the number of distant encounters, and *P*_*di*_ being the probability of infection for distant encounters. Close encounters can represent sustained contact with another individual within a household or among close friends. Distant encounters can represent an individual’s risk of catching an infection through indirect contact such as visits to supermarkets, fomites transmission or whilst commuting. The details of these two parameters can be modified to account for specific modes of transmission.

We use data from two case studies of COVID-19 transmission in Wenzhou, China [23] and Bavaria, Germany [20] to estimate parameters. We also ensure that resulting reproductive number *R*_0_ (as calculated from eq. 6) are consistent with the range estimated for COVID-19 [15, 19]. We use *n*_*ci*_ ∈ [0*,*4], *P*_*ci*_ = 0*.*4 ± 0*.*1, *n*_*ci*_ ∈ [5, 15], and *P*_*ci*_ = 0*.*025 ± 0*.*005. The number of close and distant encounters are comparable to those used for an agent-based simulation of influenza outbreaks in New York City [10]. The number of encounters and probability of infection transmission can be reduced through lockdowns and social distancing measures.

Infections begin by a period of disease incubation and are followed by a period of symptoms (unless the infection is asymptomatic). We use a mean incubation period of 5*.*1 days and a mean duration of symptoms of 11 days [17, 18, 25]. A proportion *P*_*A*_ of infections begin as asymptomatic and the remaining 1 − *P*_*A*_ begin as mild. The proportion of asymptomatic COVID-19 patients has been estimated at around 20% through airport screenings [26], on the Diamond Princess cruise ship [21] and in China [27]. We use *P*_*A*_ = 0*.*2 ± 0*.*05. Asymptomatic infected individuals may infect others, but do not change state until they recover. Mild cases have a *P*_*S*_ chance of transitioning to severe cases over the course of their illness. We use a definition of severe cases that combines the ‘severe’ and ‘critical’ categories from Wu and McGoowan [16], resulting in *P*_*S*_ = 0*.*19 ± 0*.*02. Each severely ill individual has a probability *P*_*D*_ of dying. The expected probability of dying for any infected individual, termed infection fatality rate *P*_*IFR*_ is thus:

3 $${P}_{IFR}=\left(1-{P}_A\right)\ {P}_S\ {P}_D$$

We use COVID-19 infection mortality rates from a compilation of Chinese data [16, 17, 28], calculated at 2.3 and 1.4% respectively. Accordingly, we define *P*_*D*_(0) = 0*.*12 ± 0*.*02, giving an approximate infection mortality rate of 1.8%. A full list of the parameters used and associated data sources is provided in supplementary Table 1.

### Geospatial data sources

**Fig. 1:**
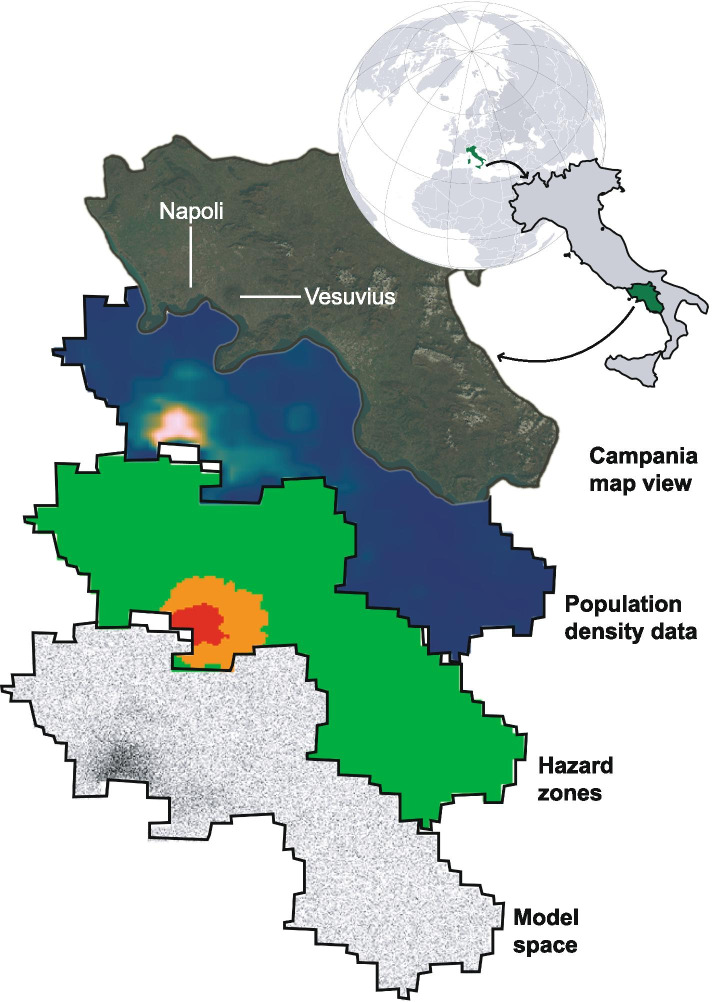
Illustration of the geospatial data used to build the geoSIR model space, using four maps of Campania. Geographical boundaries, population density data, and hazard zones are mapped onto a model grid. Satellite image from Sentinel-2. In the population density map, blue represents low population density and white represents high population density. In the hazard zones map, red, orange, and green represent the high, medium and low hazard zones, respectively. In the model space map, each black pixel represents an individual and each white pixel represents empty space

The model simulation grid is built according to real-world geospatial data. We vary the initial proportion of susceptible individuals *S* and empty space *X*_*e*_ to account for differences in population density and define the boundaries of the model space based on geographical information (Fig. 1). High and medium natural hazard areas are also inputted. These determine the areas in which evacuation may be necessary. For Vesuvius, high and medium hazard zone masks are created based on the ‘red’ and ‘yellow’ zones defined in the most recent Vesuvius National Emergency Plan [29]. Individuals living in high or medium hazard zones are tagged and retain more social interactions (e.g. a higher number of close or distant encounters) than other individuals following evacuation. Social distancing is challenging both during evacuation and in densely packed evacuation facilities [30–32].

### Model scenarios: theoretical region

We run two different experiments, one based on a theoretical region to evaluate the effect of different disaster timings and durations in the absence of real-world complexity and a second based on the region of Campania, Italy.

The theoretical region includes an idealised town or city with high population densities that radially decay away from the town centre. Initial effective population densities vary from 1 for the centre of town to 0.3 in rural areas. A concentric geological hazard is located within this study area, with the medium hazard region overlapping moderate population density outskirts of the city and rural areas. Hazard *N*_*H*_ is divided into high and medium hazard zones, with the high hazard zone entirely enveloped by the medium hazard zone. The theoretical region has a population of 100,000 individuals. We find that a population of 100,000 is sufficiently large to accurately reproduce disease spread, yet low enough to remain computationally efficient. We test 35 different scenarios, described in detail in supplementary Table 2. These scenarios vary the presence or absence of lockdown measures, the timing and duration of the natural disaster, and the type of evacuation. We conduct 100 different runs for each scenario.

### Model scenarios: Eruption of Vesuvius in Campania, Italy

Campania is Italy’s third most populated region and is home to its third largest city (Napoli). It is also home to active volcano Vesuvius. For the Campania model runs, we simulate the full 5.8 million inhabitants for 6 different scenarios. Due to the large population size in this scenario, we adapt the code to run on a 24 core Haswell E5-2680v3 processor node of the Minnesota Supercomputing Institute. One of these scenarios is comparable to reality, with no natural disaster and a lockdown implemented. We evaluate the results of it against real-world data from the wave of COVID-19 infections in spring 2020. The other five scenarios are counterfactual scenarios- one in which no natural disaster occurs but no lockdown is implemented, and the other four in which a natural disaster occurs on day 2, 25, 50 or 100 of the simulation. We use a real-world natural disaster, the eruption of Vesuvius. The key mitigation strategy for a volcanic eruption at Vesuvius is a timely evacuation [33, 34], for which evacuation plans have been designed [29]. This disaster response results in widespread population displacement - a key risk factor in disease spread [2].

## Results

**Fig. 2:**
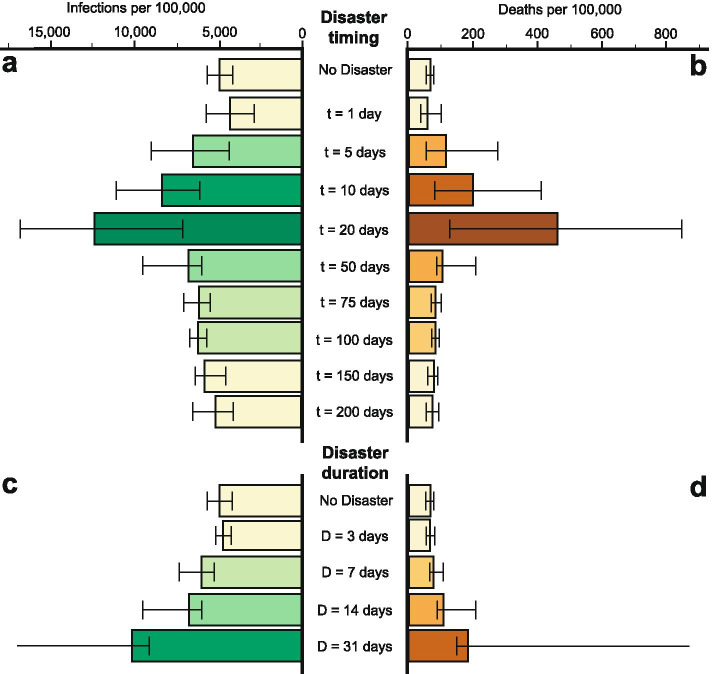
Number of infections and deaths for different theoretical disasters. The timing (**a** and **b**) and duration (**c** and **d**) of the ND are varied. Natural disasters occuring close to the peak of infections (around 20–30 days) have the largest impact. The increase in infections and deaths increases non-linearly with the duration of the disaster

In the theoretical region, we perform 100 model runs for 35 different scenarios. These scenarios cover various permutations of lockdown, natural disaster timing and natural disaster duration. Both the number of infections and number of deaths are higher when a natural disaster occurs during the infection outbreak (Fig. 2). The scale of this increase depends on the relative timing of the natural disaster and peak in infection cases, and on the duration of the disaster (Fig. 2). In the case with no natural disaster, the median number of infections is 4900 (IQR 41405,640) cases per 100,000 and the median number of deaths is 66 (IQR 53–76) per 100,000. The increase in deaths remains low when the natural disaster occurs during the onset of the outbreak (day 1, median 57, IQR 39–100 deaths per 100,000) and after the initial infection peak has subsided (day 200, median 73, IQR 53–92.5 deaths per 100,000 respectively). The number of infections and deaths is highest when the natural disaster occurs close to the peak of the outbreak. In this case, the number of infections are 153% higher and deaths are 602% higher when compared to the scenario with no natural disaster (NDt = day 20, median 12,390, IQR 7100–16,820 cases per 100,000; median 463, IQR 126–847 deaths per 100,000).

For a given natural disaster timing (50 days), long disruptions cause more infections and deaths than short ones. A 3 day long natural disaster results in a similar number of deaths (median 66.5, IQR 55–80.5 per 100,000) as the case with no natural disaster. A 31 day long natural disaster, however, causes and 180% increase in median number of deaths (median 183.5 IQR 149.5–3172 deaths per 100,000). In the case where no lockdown is instigated, around 80% of the population is infected and death toll is extreme (median 3028, IQR 2674–3259 deaths per 100,000).

For Campania, we first model the COVID-19 outbreak in a scenario similar to reality (lockdown initiated, no Vesuvius eruption). Our model predicts a median of 580 (IQR 553–641) infections per 100,000, and a median of 8.5 (IQR 7.9–8.9) deaths per 100,000. Model infections are lower, but consistent with Vollmer et al., (2020)‘s estimate of 590–950 cases per 100,000 [35] and model deaths are consistent with real world data (7.7 deaths per 100,000 in September 2020) [1].

**Fig. 3:**
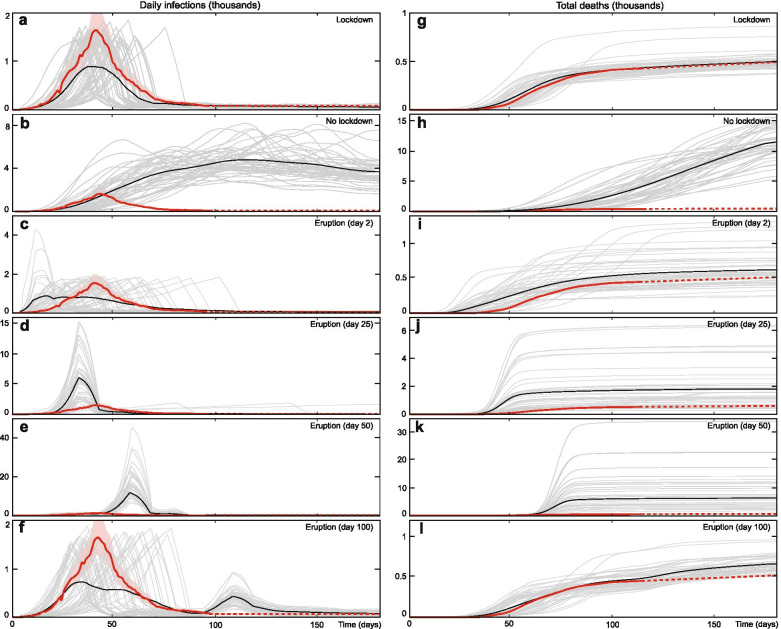
Results of the number of daily infections and deaths from COVID-19 in Campania, Italy under different scenarios. The red line represents real world data, the black line represents model mean and the grey lines represent individual model runs. All values are filtered with a 10 day moving mean to remove short period noise. The in the scenario with no eruption and a lockdown, model outputs are close to the observed real world data (**a** and **g**). Note that the model mean new daily cases appear artificially low due to different peak timings, but that the magnitude of individual runs are comparable to the real world outputs. The mean number of deaths is higher in all scenarios in which Vesuvius erupts (**c** and **i**, **d** and **j**, **e** and **k**, **f** and **l**), and higher by more than an order of magnitude where the eruption coincides with the peak in infections (**e** and **k**)

We then model five counterfactual scenarios, four of which include an eruption of Vesuvius (Fig. 3) during Campania’s COVID-19 outbreak. The increase in total number of deaths remains low for an early (Day 2, mean 9.6, IQR 7.6–13.1 deaths per 100,000) or post infections peak (Day 100, median 10.9, IQR 10–12.1 deaths per 100,000) Vesuvius eruption. The median deaths rise to.

68.5 (IQR 12.1–133.3) per 100,000 when a Vesuvius eruption occurs close to the infection peak (Day 50). In this case, the deaths per 100,000 are comparable to the states in Northern Italy worst hit by COVID-19 (168, 95 and 44 deaths per 100,000 in Lombardy, Piedmont and Veneto respectively in September 2020) or other hard-hit regions such at New York State, USA (168 deaths per 100,000 in September 2020) [1]. For an eruption occurring close to the peak in infections at day 50, there is a large variability in possible outcomes. The median death rate is 8 times higher than with no eruption (68.5 deaths per 100,000). However, in 32% of runs the death rate is no more than double that of the no eruption scenario, while in 12% of runs the death rate is more than 25 times higher.

## Discussions

Italy was one of the most hard-hit countries during the COVID-19 pandemic [7], with over 39,000 deaths and 750,000 confirmed cases as of November 2020. Campania had more than 4800 confirmed infections and 400 deaths [1]. Although the probability of Vesuvius erupting during the COVID-19 pandemic is low, the wealth of volcanic evacuation plans and COVID-19 data make it a valuable case study. The exact nature of the geological hazard is not of primary importance, rather the parameters of the evacuation and increased human contact following influence the progression of the infectious disease outbreak. The Campania results may be used to understand the effect of other disasters requiring widespread evacuation during a pandemic.

Natural disasters have already occurred during the COVID-19 pandemic [36, 37], and will occur during future infectious disease outbreaks. Cyclone Amphan made landfall in Bangladesh and West Bengal, India on May 20, 2020, prompting widespread evacuations. The number of new COVID-19 infections in the first week of June was 3.5 times higher in West Bengal and 4.8 times higher in Bangladesh compared to the same period in May [1]. The number of COVID-19 related deaths rose by a similar percentage. Further research is required to determine whether Cyclone Amphan played a role in this increase in infections and deaths, which at this stage cannot be attributed to any particular cause.

**Fig. 4:**
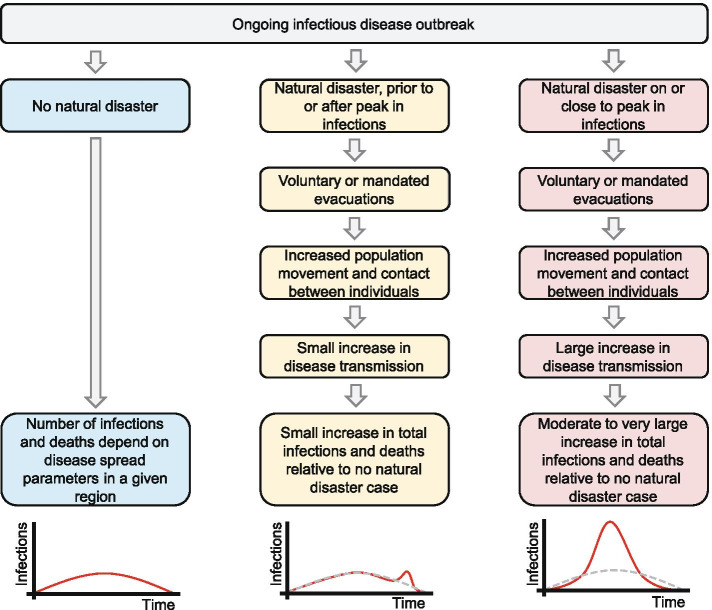
Summary of impact of natural disasters on disease outbreak, as modelled in this study

The majority of previous studies on the relationship between infectious disease outbreak and natural disasters have focused on whether natural disasters initiate new outbreaks. In a small number of cases, a disease outbreak has followed a natural disaster [2]. However, a review of the topic concluded that “the risk for epidemics after a geophysical disaster is very low” [4]. Our results do not contradict this but highlight the previously overlooked extreme risk in cases of already widespread infection (Fig. 4).

The possibility of natural hazards interacting with COVID-19 has previously been raised [3, 36, 37]. Quigley et al., 2020 use a phenomenological model to investigate the impact of natural disaster occurrence during the COVID-19 pandemic. They also find that this results in a larger total number of infections, although the simplicity of their model precludes detailed interpretation [36]. Phillips et al., 2020 propose that climate change is increasing the magnitude and frequency of climatic hazards, therefore raising the risk of natural disasters co-occurring with disease outbreaks [37].

## Conclusion

We use a stochastic SIR type model, built with a real-world geographic model space, to investigate the effect on natural disaster evacuations on the spread of COVID-19. We consider two scenarios, one in an idealised region, and one based on the region of Campania, Italy. Our model results show that in most cases, the occurrence of a natural disaster during the pandemic increases COVID-19 spread. Furthermore, we investigate the key risk factors involved in this increase and highlight the timing and duration of the natural disaster as they key controls on the increase in infections.

In our Campania test case, we model the effect of evacuation in response to an eruption of Vesuvius volcano on all 5.8 million residents, based on an existing local disaster plan. We find that an eruption occurring close to the peak in COVID-19 infections could increase the number of disease-related deaths from 8.5 (IQR 7.9–8.9) deaths per 100,000 to 68.5 (IQR 12.1–133.3) per 100,000 due to the large population displacement required in evacuating high volcanic-risk areas. The stochastic modelling approach highlights a large variability in possible outcomes for the same initial conditions, complicating disease forecasting. Close links between the epidemiology response and natural disaster response communities are necessary for the formulation of timely risk assessments.

## Supplementary information

**Additional file 1: Supplementary Table 1.** Model Parameters.

**Additional file 2: Supplementary Table 2.** Theoretical region scenarios.

**Additional file 3: Supplementary Table 3.** Campania scenarios.

## Data Availability

The code and data underlying this article are available on Zenodo, at 10.5281/zenodo.4033132 .

## Abbreviations

COVID-19: Coronavirus disease caused by SARS-COV-2 (Severe Acute Respiratory Syndrome Coronavirus 2)
IQR: Interquartile range
