# Supplementary material for: The impact of natural disasters on the spread of COVID-19: a geospatial, agent-based epidemiology model
Source: Theor Biol Med Model. 2021 Dec 3;18:20. doi: 10.1186/s12976-021-00151-0 (PMC8641790; doi:10.1186/s12976-021-00151-0)
Supplement: Supplementary file 1 — Additional file 1: Supplementary Table 1. Model Parameters. [file 12976_2021_151_MOESM1_ESM.docx]

Supplementary Table 1: Model Parameters

| **Parameters** | **Value** | **Unit** | **Source** |
| --- | --- | --- | --- |
| **Number of people (Campania)** | 5800000 | persons | Population data |
| **Number of close encounters** | 0 to 4 | Encounters per day | Bomer et al., 2020 |
| **Probability of infection for close encounters** | 40±10 | % | Cai et al., 2020 |
| **Number of distant encounters** | 5 to 15 | Encounters per day | Bomer et al., 2020 |
| **Probability of infection for distant encounters** | 2.5±0.5 | % | Cai et al., 2020 |
| **Number of travellers in** | 10 | Persons | N/A |
| **Number of travellers out** | 10 | Persons | N/A |
| **Risk of serious illness** | 19±2 | % | Wu and McGoogan, 2020 |
| **Proportion of asymptomatic** | 20±5 | % | Henegan et al., 2020 |
| **Death rate of seriously ill** | 12±2 | % | Wu and McGoogan, 2020;  Guan et al., 2020 |
| **Hospital capacity**  **(Campania)** | 427 | ICU beds | Pecoraro et al., 2020 |
| **Incubation time** | 5.1±2.5 | Days | Lauer et al., 2020; Guan et al., 2020 |
| **Duration of symptoms** | 11±2 | Days | Zhou et al., 2020 |
| **Threshold for lockdown** | If confirmed cases of infections reach 2000 **OR** number of deaths reach 50 | Persons | N/A |
| **Threshold for travel ban** | If confirmed cases of infections reach 500 **OR** number of deaths reach 50 | Persons | N/A |
