# Supplementary material for: The impact of natural disasters on the spread of COVID-19: a geospatial, agent-based epidemiology model
Source: Theor Biol Med Model. 2021 Dec 3;18:20. doi: 10.1186/s12976-021-00151-0 (PMC8641790; doi:10.1186/s12976-021-00151-0)
Supplement: Supplementary file 2 — Additional file 2: Supplementary Table 2. Theoretical region scenarios. [file 12976_2021_151_MOESM2_ESM.docx]

Supplementary table 2: Theoretical region scenarios

| **Scenario number** | **Lockdown?** | **Natural disaster?** | **Disaster timing (days)** | **Disaster duration (days)** | **Evacuation type*** |
| --- | --- | --- | --- | --- | --- |
| 1 | No | No | - | - | - |
| 2 | Yes | No | - | - | - |
| 3 | Yes | Yes | 1 | 14 | 4 |
| 4 | Yes | Yes | 5 | 14 | 4 |
| 5 | Yes | Yes | 10 | 14 | 4 |
| 6 | Yes | Yes | 20 | 14 | 4 |
| 7 | Yes | Yes | 50 | 14 | 4 |
| 8 | Yes | Yes | 75 | 14 | 4 |
| 9 | Yes | Yes | 100 | 14 | 4 |
| 10 | Yes | Yes | 150 | 14 | 4 |
| 11 | Yes | Yes | 200 | 14 | 4 |
| 12 | Yes | Yes | 5 | 3 | 4 |
| 13 | Yes | Yes | 20 | 3 | 4 |
| 14 | Yes | Yes | 50 | 3 | 4 |
| 15 | Yes | Yes | 100 | 3 | 4 |
| 16 | Yes | Yes | 5 | 7 | 4 |
| 17 | Yes | Yes | 20 | 7 | 4 |
| 18 | Yes | Yes | 50 | 7 | 4 |
| 19 | Yes | Yes | 100 | 7 | 4 |
| 20 | Yes | Yes | 5 | 31 | 4 |
| 21 | Yes | Yes | 20 | 31 | 4 |
| 22 | Yes | Yes | 50 | 31 | 4 |
| 23 | Yes | Yes | 100 | 31 | 4 |
| 24 | Yes | Yes | 5 | 14 | 1 |
| 25 | Yes | Yes | 20 | 14 | 1 |
| 26 | Yes | Yes | 50 | 14 | 1 |
| 27 | Yes | Yes | 100 | 14 | 1 |
| 28 | Yes | Yes | 5 | 14 | 2 |
| 29 | Yes | Yes | 20 | 14 | 2 |
| 30 | Yes | Yes | 50 | 14 | 2 |
| 31 | Yes | Yes | 100 | 14 | 2 |
| 32 | Yes | Yes | 5 | 14 | 3 |
| 33 | Yes | Yes | 20 | 14 | 3 |
| 34 | Yes | Yes | 50 | 14 | 3 |
| 35 | Yes | Yes | 100 | 14 | 3 |

*1: Increased movement of people and lifted lockdown in high and medium hazard zones, no evacuation

2: Evacuation of people only in high-hazard zone, no increase in movement

3: Evacuation of people in high-hazard zone, increase in movement in medium and high hazard zones

4: Evacuation of high hazard zone, 25% of individuals in medium hazard zone and increase in movement in medium, high and low hazard zone (e.g. Lu et al., 2012; doi.org/10.1073/pnas.1203882109)
