# Supplementary material for: The impact of natural disasters on the spread of COVID-19: a geospatial, agent-based epidemiology model
Source: Theor Biol Med Model. 2021 Dec 3;18:20. doi: 10.1186/s12976-021-00151-0 (PMC8641790; doi:10.1186/s12976-021-00151-0)
Supplement: Supplementary file 3 — Additional file 3: Supplementary Table 3. Campania scenarios. [file 12976_2021_151_MOESM3_ESM.docx]

Supplementary table 3: Campania scenarios

| **Scenario number** | **Lockdown?** | **Natural disaster?** | **Disaster timing (days)** | **Disaster duration (days)** |
| --- | --- | --- | --- | --- |
| 1* | Yes | No | - | - |
| 2 | No | No | - | - |
| 3 | Yes | Yes | 2 | 14 |
| 4 | Yes | Yes | 25 | 14 |
| 5 | Yes | Yes | 50 | 14 |
| 6 | Yes | Yes | 100 | 14 |

*Scenario 1 is the closest to the real scenario in Campania, through early 2020.
